# THE ROLE OF SOCIAL NETWORK CHARACTERISTICS IN LONG-TERM CARE NETWORKS AMONG OLDER ADULTS IN THE NETHERLANDS

**DOI:** 10.1093/geroni/igae098.0120

**Published:** 2024-12-31

**Authors:** Jens Abbing, Joukje Swinkels, Marjolein Broese Van Groenou

**Affiliations:** Vrije Universiteit Amsterdam, Amsterdam, Noord-Holland, Netherlands; VU Amsterdam, Amsterdam, Noord-Holland, Netherlands; Vrije Universiteit Amsterdam, Amsterdam, Noord-Holland, Netherlands

## Abstract

Objectives The increasing demand for long-term care (LTC) among western societies calls for a better understanding of how older adults build LTC networks consisting of informal and formal caregivers. Having supportive relationships is generally associated with receiving informal care, yet how specific characteristics of individuals’ social networks relate to LTC networks is not fully understood. Therefore, the present study investigated how the size, composition, and relationship quality of individuals’ social network influences what type of LTC network they have. Methods Data was used from the Longitudinal Aging Study Amsterdam that measured LTC use and social network characteristics of older adults (average age=70.88) in three-year intervals from 1992 to 2022. The sample contained 18,252 complete observations from 4,878 participants. Results Using multilevel latent class analysis, six LTC network types characterized by the main caregiver (co-residents, children, relatives, non-kin, publicly paid care and privately paid care) were identified. Cluster-robust multinomial logistic regression analyses revealed that social network size, composition and relationship quality all were associated with membership in the various informal care network types. In particular having a partner, and children and relatives living in proximity and/or with strong bonds contributed to an informal LTC network. Discussion Network composition was most important while the impact of relationship quality was relatively small. This suggests that older adults may refrain from using the full potential of their social network to build informal care networks, possibly because they prefer not to involve relatives or non-kin into caregiving or prefer formal or privately paid care.

